# EndoS from *Streptococcus pyogenes *is hydrolyzed by the cysteine proteinase SpeB and requires glutamic acid 235 and tryptophans for IgG glycan-hydrolyzing activity

**DOI:** 10.1186/1471-2180-8-3

**Published:** 2008-01-08

**Authors:** Maria Allhorn, Arne Olsén, Mattias Collin

**Affiliations:** 1 Department of Clinical Sciences, Division of Infection Medicine, Lund University, Biomedical Center B14, SE-221 84 Lund, Sweden; 2 Molecular Pharmacology AstraZeneca R & D, SE-431 83 Mölndal, Sweden

## Abstract

**Background:**

The endoglycosidase EndoS and the cysteine proteinase SpeB from the human pathogen *Streptococcus pyogenes *are functionally related in that they both hydrolyze IgG leading to impairment of opsonizing antibodies and thus enhance bacterial survival in human blood. In this study, we further investigated the relationship between EndoS and SpeB by examining their *in vitro *temporal production and stability and activity of EndoS. Furthermore, theoretical structure modeling of EndoS combined with site-directed mutagenesis and chemical blocking of amino acids was used to identify amino acids required for the IgG glycan-hydrolyzing activity of EndoS.

**Results:**

We could show that during growth *in vitro S. pyogenes *secretes the IgG glycan-hydrolyzing endoglycosidase EndoS prior to the cysteine proteinase SpeB. Upon maturation SpeB hydrolyzes EndoS that then loses its IgG glycan-hydrolyzing activity. Sequence analysis and structural homology modeling of EndoS provided a basis for further analysis of the prerequisites for IgG glycan-hydrolysis. Site-directed mutagenesis and chemical modification of amino acids revealed that glutamic acid 235 is an essential catalytic residue, and that tryptophan residues, but not the abundant lysine or the single cysteine residues, are important for EndoS activity.

**Conclusion:**

We present novel information about the amino acid requirements for IgG glycan-hydrolyzing activity of the immunomodulating enzyme EndoS. Furthermore, we show that the cysteine proteinase SpeB processes/degrades EndoS and thus emphasize the importance of the SpeB as a degrading/processing enzyme of proteins from the bacterium itself.

## Background

Extracellular enzymes from *Streptococcus pyogenes *have been extensively studied and shown to be of importance for the pathogenesis of this human pathogen (for a review see [1]). The secreted *S. pyogenes *enzyme EndoS (AAK00850) has a specific endoglycosidase activity on native human IgG by hydrolyzing the conserved asparagine-linked glycans found on each heavy chain of IgG [2]. EndoS-activity affects the functionality of opsonizing IgG by decreased binding to Fc-receptors and impaired classical complement activation, and EndoS treatment of human opsonizing IgG antibodies directed towards the cell-wall anchored M protein significantly enhances bacterial survival in human blood [3]. The *ndoS *gene encoding EndoS is present in all tested isolates, and is highly conserved. Both healthy and infected humans have circulating antibodies against EndoS, suggesting *in vivo *expression [4]. In addition, EndoS is up regulated when interacting with white blood cells [5]. The activity of EndoS on IgG may be beneficial for *S. pyogenes *expressing this enzyme with modulation and/or evasion of an IgG-mediated response against the bacteria. In contrast to this, the purified form of EndoS has substantial potential as a therapeutical agent against antibody-mediated autoimmune diseases and other conditions where IgG is involved in pathological processes. It has recently been shown that pre-treatment of arthritogenic IgG antibodies with EndoS abrogates development of arthritis in a mouse model of collagen-induced arthritis [6].

One of the most studied streptococcal enzymes is the cysteine proteinase, SpeB. Several *in vitro *and *in vivo *studies, as well as clinical studies have suggested a role for SpeB as an important virulence factor [7-9]. SpeB has the ability to degrade the human extracellular matrix protein fibronectin and vitronectin, release inflammatory mediators such as interleukin 1β and bradykinin from their precursors, cleave or degrade immunoglobulins and complement factors, and also bind to the human cell surface receptors integrins [10-17]. In addition, SpeB releases active fragments from cell wall-anchored proteins from the bacterium itself, cleaves the secreted pore-forming streptolysin O that retains its cytolytic activity after processing, and degrades superantigens [18-20].

EndoS and SpeB from *S. pyogenes *are functionally related in that they both hydrolyze IgG leading to impairment of opsonizing antibodies and thus enhance bacterial survival in human blood [3].

In this study, we further investigated the relationship between EndoS and SpeB by examining their *in vitro *temporal production and reveal a novel activity of SpeB; processing and eventually complete degradation of EndoS with loss of its IgG hydrolyzing activity. Furthermore, theoretical structure modeling of EndoS combined with site-directed mutagenesis and chemical blocking of amino acids identified amino acids required for the IgG glycan-hydrolyzing activity of EndoS.

## Results and Discussion

### Temporal production of EndoS and SpeB

**Figure 1:**
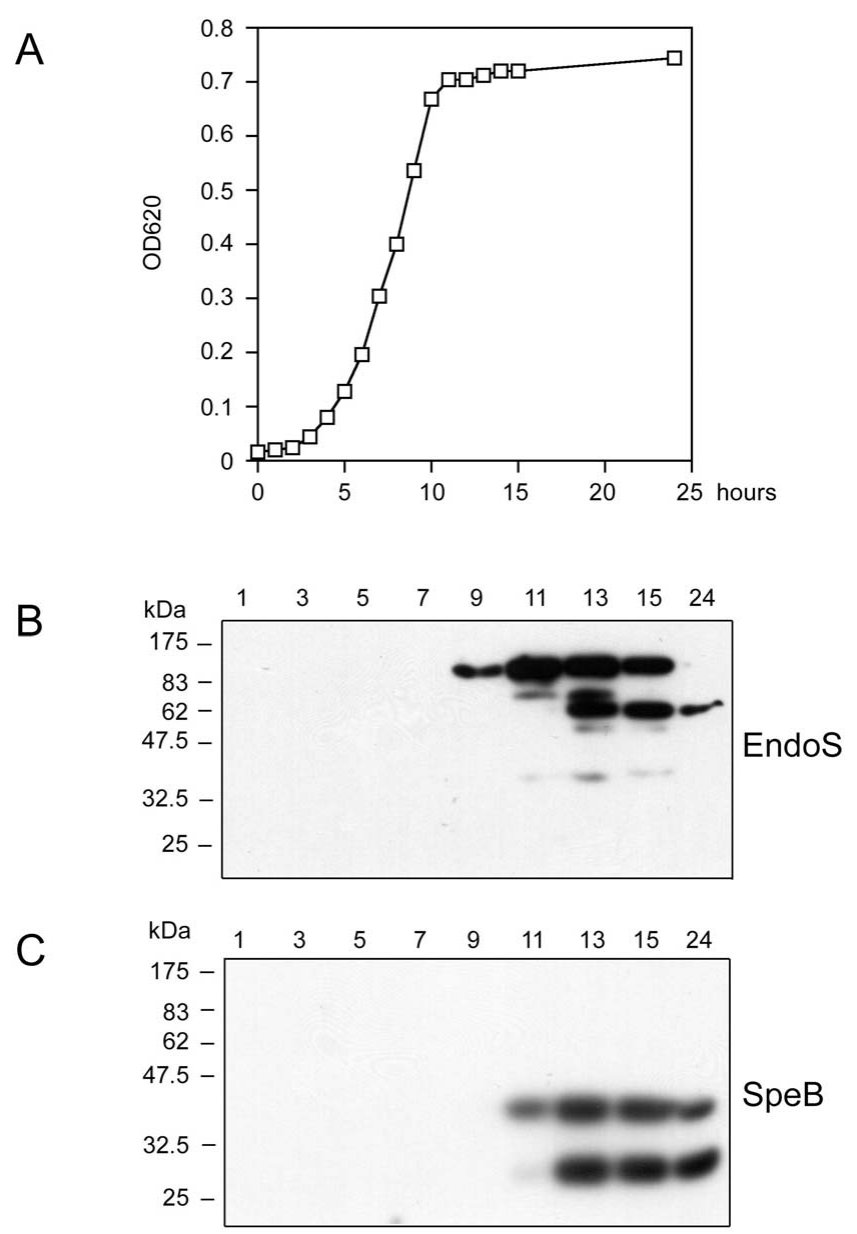
**Secretion of EndoS and SpeB during growth of *S. pyogenes *strain AP1**. Panel A, growth of wild type AP1 and measurements of optical density at 620 nm over time. Samples were withdrawn for analysis at indicated time points. Panel B, detection of EndoS in samples from indicated time points using antiserum against full-length EndoS. Panel C, detection of SpeB in the culture supernatants using antiserum against the 40 kDa zymogen form of SpeB.

*S. pyogenes *strain AP1 was cultured in a medium for optimal expression of EndoS and SpeB [2,21], and culture supernatant samples were withdrawn at indicated time points (Fig. 1A). Secretion of SpeB and EndoS was analyzed using Western blots with polyclonal rabbit antiserum raised against the zymogen form of SpeB and full-length EndoS. Expression of intact EndoS (108 kDa) was observed after approximately 9 hours in late exponential growth phase (Fig. 1A and 1B). However, when samples were collected at 12 hours of incubation an additional band with an apparent mass of 62 kDa reacted with the EndoS antibodies (Fig. 1A and 1B). Furthermore, the total amount of the 62 kDa band decreased between 15 and 24 hours of culturing suggesting further degradation of this fragment of EndoS. Interestingly, the appearance of this 62 kDa EndoS fragment coincides with the maturation and processing of SpeB from its 40 kDa zymogen form into its proteolytically active 28 kDa form (Fig. 1C). This observation suggested that SpeB is involved in the processing/degradation of EndoS, and we hypothesized that this could be of importance in regulating EndoS activity.

### SpeB hydrolyzes EndoS during growth and in purified form

**Figure 2:**
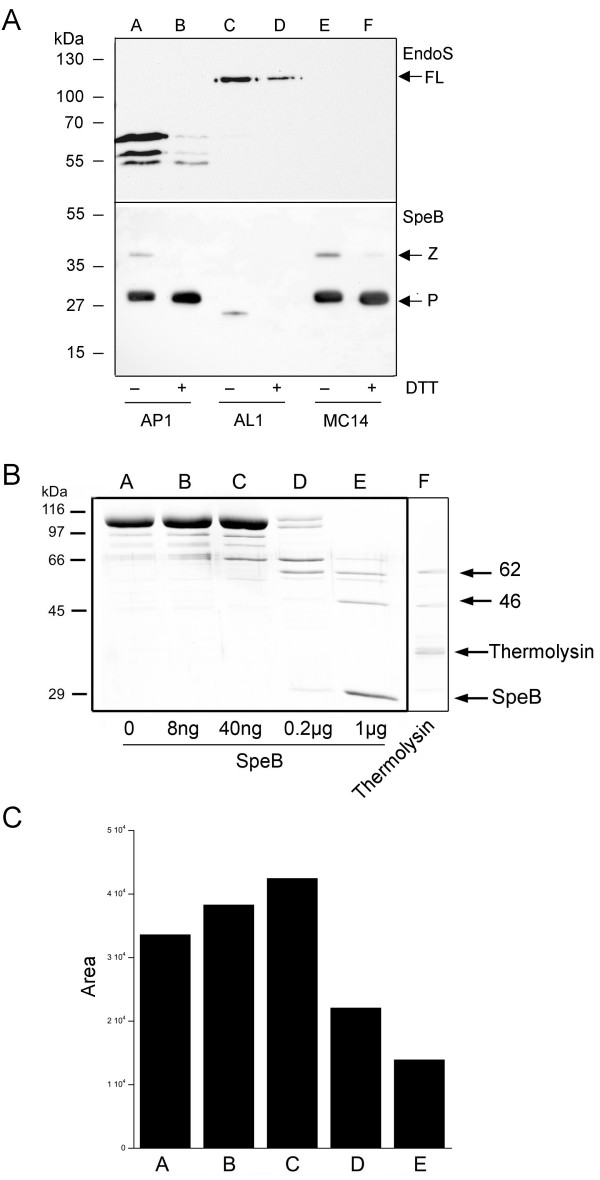
**EndoS is hydrolyzed by SpeB**. Panel A, analysis of SpeB-hydrolysis of EndoS during growth of the wild type AP1 (lanes A and B), the SpeB mutant AL1 (lane C and D), and the EndoS mutant MC14 (lanes E and F) with antiserum against EndoS and SpeB. Presence or absence of DTT during growth is indicated under the lower panel. Arrows to the right indicate the positions of full-length EndoS (FL), the zymogen (Z) and proteinase (P) forms of SpeB. Panel B, rEndoS incubated with increasing amounts of SpeB (lanes A-E) or thermolysin (lane F) as indicated and analyzed by SDS-PAGE. Arrows to the right indicate the positions of the 46- and **62**-kDa forms of EndoS, SpeB, and thermolysin. Panel C, densitometric analysis of whole lanes A-E (excluding SpeB) in panel B. Values are presented as area under the curve in pixels.

In order to confirm that SpeB hydrolyzes EndoS, we cultured the wild type strain AP1 for 20 hours in CM with or without the reducing agent DTT (SpeB activity requires reducing conditions [22]) and analyzed the status of EndoS and SpeB using Western blots. Under reducing conditions the AP1 strain nearly completely degraded EndoS protein, while under the slightly reducing condition in CM alone EndoS was only partly hydrolyzed (Fig. 2A, EndoS, lanes A and B), which suggests that SpeB is responsible for the hydrolysis of EndoS. Analysis using SpeB antibodies revealed that under non-reducing conditions the AP1 strains produces mainly the 28 kDa active form of SpeB, but some 40 kDa could be detected, while under reducing conditions only the active form of SpeB could be detected (Fig 2B, SpeB, lanes A and B). The 62 kDa band reacting with the EndoS antibodies in lane A figure 2A was sequenced by Edman degradation [23] revealing the sequence KDKSYDLI corresponding to amino acids 446–453 of the EndoS sequence (AAK00850). Thus, one SpeB cleavage site in EndoS is between Leu-445 and Lys-446, but smaller degradations products reacting with the antibodies could also be seen. To confirm SpeB activity on EndoS, we cultured the isogenic SpeB mutant AL1 [2,24] in CM and analyzed the supernatant as above. This showed that AL1 is unable to hydrolyze EndoS even in the presence of DTT (Fig. 2A, EndoS, lanes C and D). A confirmation that AL1 does not produce any active SpeB is seen in (Fig. 2B, SpeB, lanes C and D). As a control experiment, strain MC14 mutated in the *ndoS *gene was analyzed under the same conditions. This revealed that MC14 does not produce any EndoS (Fig. 2A, EndoS lanes E and F), while it still produces SpeB as wild type AP1 (Fig. 2B, SpeB, lanes E and F). Taken together, SpeB cleaves EndoS into a main 62 kDa fragment and several smaller fragments during growth, but it remained unclear whether the remainder of EndoS was completely degraded or simply not recognized by the EndoS-antibodies.

To further investigate the hydrolysis of EndoS, recombinantly expressed EndoS (rEndoS) was incubated with serial dilutions of SpeB. This revealed that SpeB processes rEndoS into two major fragments of approximately 62 and 46 kDa (Fig. 2B, lane E). Visual inspection and whole lane densitometry analysis (Fig 2C) showed that the total amount of rEndoS also diminished, indicating that besides processing into two fairly stable fragments, SpeB unspecifically degrades EndoS into low molecular weight fragments. In addition, EndoS was incubated at an enzyme: substrate ratio of 1:3 with thermolysin for one hour. Despite 296 predicted thermolysin sites in the mature 108 kDa form of EndoS, two major fragments of approximately 62 and 46 kDa, similarly to what could be seen with SpeB, resisted proteolysis (Fig. 2B, lane F).

### Analysis of primary structure and homology modeling of EndoS

**Figure 3:**
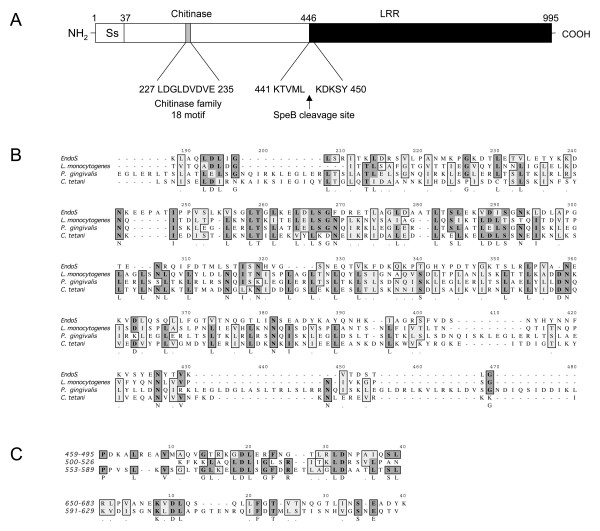
**Possible domain organization and sequence alignments of EndoS**. Panel A, schematic representation of the 995 amino acids EndoS. Ss indicates signal peptide, the chitinase family 18 active site motif in the amino-terminal domain is indicated, the SpeB cleavage site is indicated with an arrow, and the putative leucine-rich repeat region (LRR) is shown. Panel B, ClustalW alignment of EndoS and leucine-rich proteins from *P. gingivalis *(NP_905954), *C. tetani *(NP_781184), and *L. monocytogenes *(NP_463795). Amino acid number one corresponds to amino acid 446 in the whole protein. Panel C, RADAR repeat analysis showing three 37 amino acids leucine rich repeats and two additional repeats. Numbering is based on the 995 amino acids sequence of full-length EndoS.

EndoS contains a 37 amino acids N-terminal signal sequence that has been verified by amino-terminal sequencing [2], an amino-terminal part comprising amino acids 37–446 which harbors a family 18 glycosyl hydrolase active site motif at position 227–235 [25] (Fig. 3A). The remaining part (amino acids 446–995) of EndoS carboxy-terminally to the SpeB cleavage site (Fig. 3A. 446–995) is similar to leucine-rich repeat proteins (LRR's) from the oral pathogen *Porphyromonas gingivalis *[26], the tetanus-causing *Clostridium tetani *[27], and from the intracellular pathogen *Listeria monocytogenes *(Internalin E, InlE) [28] (Fig. 3B). No functions have been ascribed to the LRR's most similar to EndoS, but LRR's belonging the internalin family of proteins from *Listeria spp*. are essential for cellular attachment and internalization through binding of E-cadherin [29,30]. EndoS contains three highly similar leucine-rich repeats of approximately 37 amino acids between amino acids 459 and 589, and two additional repeats between 591 and 683 with lower leucine content and with a somewhat lower similarity as detected by the RADAR algorithm [31](Fig. 3C).

**Figure 4:**
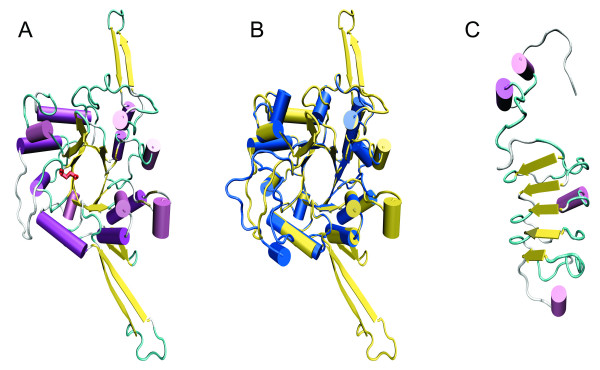
**Homology modeling of EndoS**. Panel A, model of amino acids 37–446 of EndoSusing EndoF_3 _as the template. Glu-235 in the active is shown in red. Panel B, EndoS model (yellow) superimposed in the structure of EndoF_3 _(blue). Panel C, model of amino acids 446–557 of EndoS using the LRR-region from InlB as the template. Panel A and C, β-strands are shown in yellow, α-helices in purple, and loops in turquoise.

Despite a wide variety of folds, the overall topology of glycosyl hydrolases can be divided into three main groups where the active site is within a pocket, a cleft or a tunnel. The varying degree of accessibility to the active site has been suggested to determine the carbohydrate substrate specificity [32]. Since the only substrate for EndoS known to date is the complex bi-antennary glycan on native IgG, we attempted to predict the structure of EndoS using homology modeling. Amino acids 37–446 of EndoS was aligned with EndoF_3 _from *Elizabethkingia meningoseptica *(formerly *Flavobacterium meningosepticum*) [33] and subjected to automated protein homology modeling using EndoF_3 _(PDB 1EOK) as the template. The generated model of amino acids 37–446 covers the active site and displays a typical (α/β)_8_-barrel with eight repeated β-strand/loop/α-helix units with the parallel β-strands forming an eight-stranded β-barrel (Fig. 4A). The putative catalytic Glu-235 residue in the FGH18 active site is located at the orifice of the β-barrel. When superimposing the EndoS model on the EndoF_3 _structure, two additional antiparallel β-strands protrudes at the top of the barrel close to the catalytic Glu residue, and there are two extended loops with antiparallel β-strands on opposite sides of the molecule. Both EndoS and EndoF_3 _hydrolyse biantennary complex carbohydrates [2,33]. The modeled β-barrel in EndoS potentially only accommodates certain glycans and could partly explain EndoS' specificity for the N-linked carbohydrate on human IgG, even though its activity has been suggested to depend on additional protein-protein interactions [34]. Hypothetically, the additional extended loops in the EndoS could contribute to the specificity by interactions with the protein backbone of the IgG heavy chain. Homology models should be interpreted with great caution, but the EndoS model could serve as a starting point for rational mutagenesis, interaction studies with IgG, and attempts to crystallize defined parts of the molecule.

Since EndoS contains LRR's with similarities to other bacterial LRR proteins we attempted to generate a model of this region to investigate if EndoS has the potential of adopting a similar structure known to be important for protein-protein interaction and virulence in *Listeria spp*. Using the same approach as for the amino-terminal part we were able to construct a model of amino acids 446–577 of EndoS using the LRR region of Internalin B (InlB, PDB 1M9S) from *Listeria monocytogenes *as a template [35]. This model revealed a bowed tube structure with five parallel β-strands forming a β-sheet that constitute the concave face of the structure in a similar manner as can be seen in InlB (Fig. 4C). In the EndoS-LRR model there is only one complete β-loop-helix-loop motif that can be seen in several LRR proteins including InlB. Furthermore, it is unclear from this model if the carboxy-terminal part of EndoS has the kind of cap structure that can be seen in InlB. Nevertheless, it is intriguing that a secreted *S. pyogenes *protein has structural similarity with LRR's of internalins that are crucial for cellular invasion and virulence. Furthermore, since the concave face of other LRR's including InlB has been suggested to be a major site for protein interactions, the model of the LRR region in EndoS could be used as a starting point for finding potential cellular and protein targets in the human host.

### Site-directed mutagenesis and chemical modification of tryptophans inactivates EndoS

**Figure 5:**
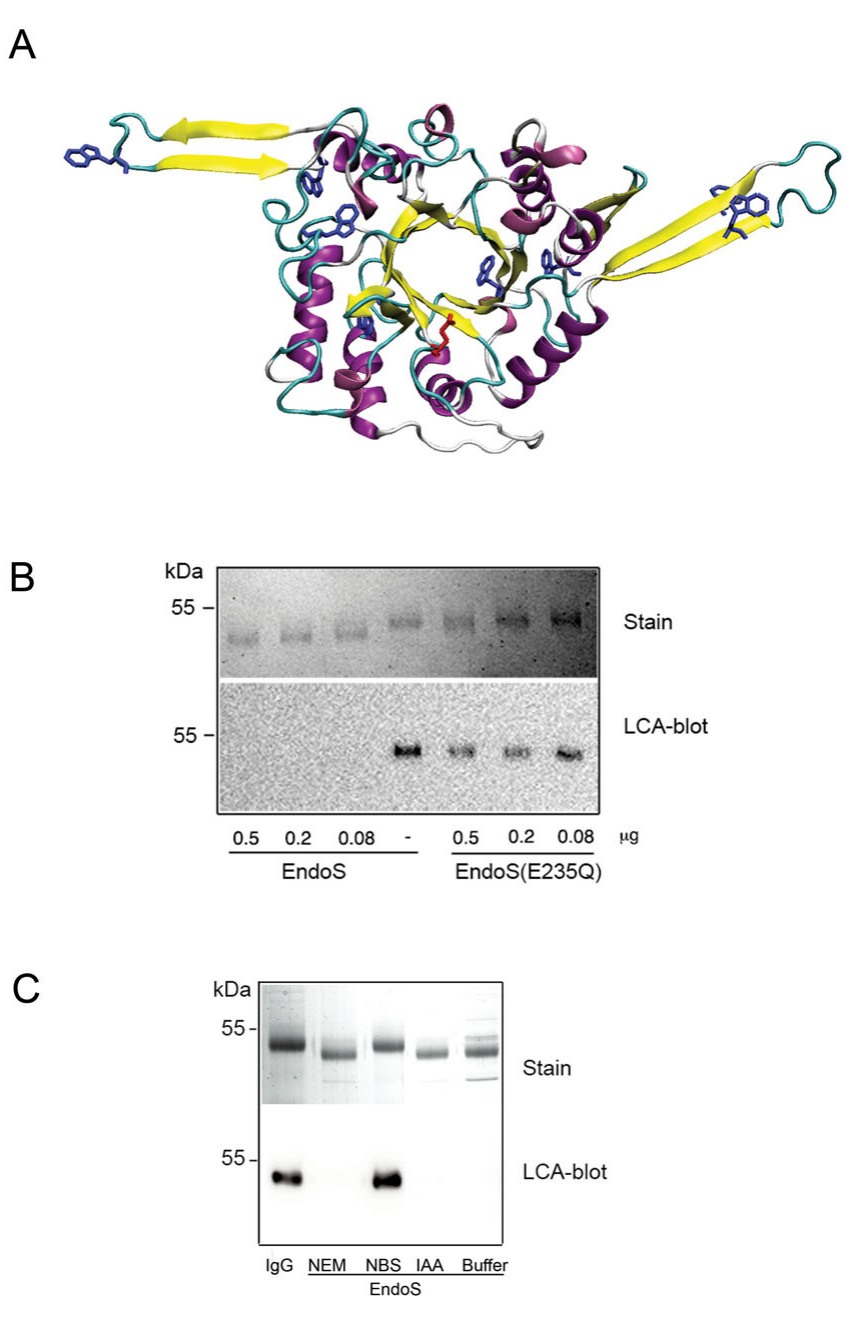
**Glutamic acid 235 and tryptophan residues are important for EndoS activity**. Panel A, homology model of the amino-terminal part of EndoS with glutamic acid 235 (red) and the tryptophan residues (blue) are highlighted. Panel B, dilutions of rEndoS or rEndoS(E235Q) incubated with 1 μg human IgG for 1 hour at 37°C. IgG-glycan hydrolysis by EndoS was analyzed by SDS-PAGE and LCA-blot. Panel C, human IgG incubated with buffer, rEndoS modified with NEM, NBS, IAA, or unmodified (Buffer). The proteins were separated by SDS-PAGE, stained or analyzed for LCA reactivity in blot.

Sequence analysis suggested that Glu-235 is part of the glycosyl hydrolase motif and possibly the catalytically active amino acid. Furthermore, the positioning of Glu-235 at the orifice of the -barrel as suggested by homology modeling identified this amino acid as prime catalytic residue candidate (Fig. 5A). Therefore we mutated Glu-235 in rEndoS to glutamine by site-directed mutagenesis of the *ndoS *gene and recombinantly expressed EndoS(E235Q). This protein was tested for activity against purified human IgG by SDS-PAGE and lectin blot analysis. This revealed that EndoS, but not EndoS(E235Q) could shift the apparent mass of the IgG heavy chain approx. 3 kDa (Fig 5B, Stain) indicating that EndoS(E235Q) has lost its activity on IgG. Furthermore, IgG heavy chains incubated with EndoS but not EndoS(E235Q) loose the reactivity against the mannose-specific biotinylated lectin *Lens culinaris *agglutinin (LCA) (Fig 5B, LCA-blot). We have previously shown that lack of lectin signal from IgG corresponds well to complete hydrolysis of the chitobiose core of the IgG-glycan as analyzed by mass spectroscopy of hydrolyzed IgG [36]. This experiment confirmed that Glu-235 is essential for the IgG-glycan hydrolyzing activity of EndoS.

Another approach to investigate which amino acids that are important for enzymatic activity involves chemical modification of certain types of amino acids. It has previously been shown that chemical blocking of tryptophans in a β-*N*-acetylglucosaminidase from the mollusk *Batillus cornutus *(formerly *Turbo cornutus*) inhibits chitinase activity [37]. Furthermore, tryptophans situated on extended loops outside the catalytic site are essential for substrate binding and enzymatic activity in a chitinase from *Streptomyces griseus *[38,39]. This was intriguing, since 8 tryptophans are present in the amino-terminal part of EndoS of which one is located inside the predicted -barrel with Glu-235 at the orifice, and three (two and one) in the predicted extended loops that distinguish EndoS from the template EndoF_3 _(Fig 5A). Furthermore, there are four more tryptophan residues in the carboxy-terminal part located carboxy-terminally to the LRR. Therefore we investigated if N-bromosuccinimide (NBS) could affect the enzymatic activity of EndoS. NBS can react with the indole group of tryptophan and with SH groups of cysteines and are commonly used for characterization amino acid residues involved in enzymatic activity [40]. After modification of EndoS with NBS, no IgG glycan hydrolysis could be seen as analyzed by SDS-PAGE and LCA lectin blot analysis (Fig. 5C, Stain and LCA-blot). Treatment of EndoS with iodoacetamide (IAA) or N-ethylmaleimide (NEM), reagents used for blockage of cysteines (Fig. 5C, Stain and LCA-blot, NEM and IAA), or modification of lysines using formaldehyde, did not affect the hydrolysis of IgG by EndoS (data not shown). These results suggest that lysine (constitutes 10% of the amino acids) and cysteine residues (one in the mature protein) in EndoS are not essential for the enzymatic activity, while tryptophans in the predicted β-barrel, in the extended loops, and/or in the carboxy-terminal part are required for the IgG glycan-hydrolyzing activity of EndoS.

### SpeB ultimately inactivates EndoS

**Figure 6:**
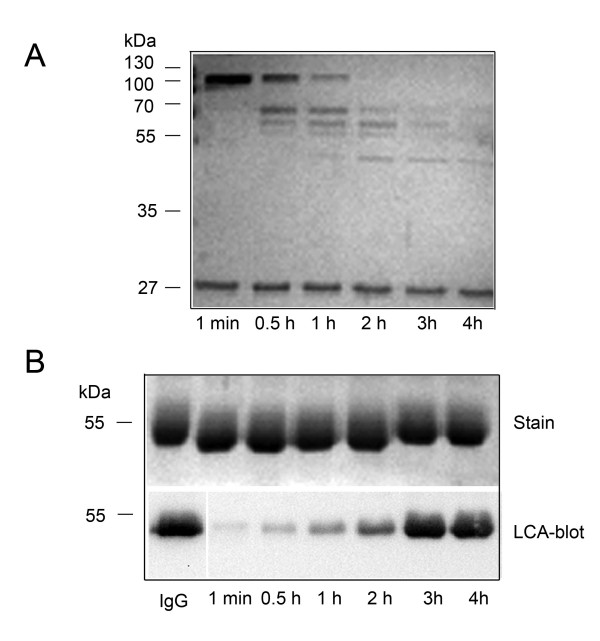
**SpeB-hydrolyzed rEndoS loses activity on human IgG**. Panel A, rEndoS was incubated with SpeB as described in material and methods at indicated time points and 10 μl of samples were applied on polyacrylamide gel and stained with Coomassie Blue. Panel B, 2 μl sample withdrawn from the same incubations, was incubated with 2 g IgG for 2 hours and IgG hydrolysis was analyzed by SDS-PAGE and LCA-blot.

To investigate whether SpeB-hydrolyzed EndoS has activity on the glycan on IgG, rEndoS was treated for increasing amount of time with SpeB prior to incubation with human IgG. This revealed that SpeB hydrolyzes EndoS in a time-dependent manner and that after 2 hours of incubation virtually no full-length EndoS is present. The smaller protein fragment can still be detected after 4 hours and the larger fragment can be detected after 3 h (Fig. 6A). This corresponds well to the observations of the presence and state of the two enzymes during growth of *S. pyogenes*; when active SpeB starts to appear after 12–13 hours of growth the first signs of EndoS-hydrolysis can be seen, and 2–3 hours later no full-length EndoS can be detected (Fig. 1 and data not shown). Furthermore, the IgG glycan-hydrolyzing activities of the same samples were analyzed by SDS-PAGE and LCA lectin blot. This revealed that after 3 hours of incubation no IgG glycan hydrolyzing activity could be detected (Fig. 6B, Stain and LCA-blot). This indicates that full-length EndoS is required for activity on IgG and that SpeB eventually inactivates the IgG glycan-hydrolyzing activity of EndoS.

In this study we show that EndoS and SpeB are coordinately expressed during growth *in vitro*, and that when SpeB maturates into its active form it starts to degrade EndoS, but two major fragments of 62 and 46 kDa seems somewhat more resistant to proteolysis. Fragments of similar sizes are also generated by the protease thermolysin suggesting that there are domains in EndoS that are partly protected against proteolysis, of which one most likely is the 62 kDa carboxy-terminal fragment identified by amino-terminal sequencing. SpeB-processing and/or degradation of proteins from the bacterium itself seem to be important mechanisms for inactivation or release of biologically active fragments or domains. SpeB processes the pore-forming streptolysin O [19], releases functional fragments from the cell-wall anchored C5a-peptidase and IgG-binding M proteins [18], and degrades the streptococcal superantigen SmeZ [20]. Such a process might be of importance in controlling the enzymatic activity of EndoS or to release fragments with biological activity, but at this point we could only substantiate the former. Interestingly, a recent study has shown in a mouse model of subcutaneous infection that *S. pyogenes *undergoes a stable phase shift to low SpeB production leading to production of intact forms of several putative and known virulence factors including EndoS [41]. This further emphasizes the role for SpeB in controlling the activity of other secreted and cell wall-anchored proteins during the infection process.

Attempts to recombinantly express the amino- and carboxy-terminal parts of EndoS separately for functional studies have this far proven unsuccessful. The amino-terminal part could be expressed in *E. coli*, but the protein is degraded despite the use of protease-deficient host strains, while the gene fragment encoding the carboxy-terminal part could not be established in *E. coli *for unknown reasons (data not shown). Furthermore, we have not been able to separate the SpeB generated fragments of EndoS using gel filtration, ion-exchange chromatography, or affinity chromatography with immobilized EndoS antibodies (data not shown).

Homology modeling, site-directed mutagenesis, and chemical modification experiments of EndoS suggest that the amino-terminal part contains several key elements necessary for enzymatic activity on human IgG. The function of the carboxy-terminal part remains unknown, but the observation that only full-length EndoS has activity on IgG indicates that also this part of the protein is important for the interaction with IgG, either as necessary structural element or by direct interactions with IgG. The primary and possible structural similarities between EndoS and LRR's from other pathogenic bacteria suggest that it might have similar adhesive or invasive functions either as a liberated domain or in the intact enzyme. Interestingly, another recently identified extracellular *S. pyogenes *LRR protein; Slr (streptococcal leucine-rich repeat protein) is involved in virulence and phagocytosis resistance [42]. Our findings may in part explain why EndoS in contrast to many related endoglycosidases, that require or are enhanced by denaturation of the glycoprotein, only interacts with native IgG [34]. A protein-protein interaction between the enzyme and IgG involving tryptophan residues in EndoS could be part of this unique feature.

It is currently not known how the IgG-hydrolyzing activities of SpeB and EndoS contributes to the pathogenesis of *S. pyogenes *infections, even though the attenuation seen in SpeB mutants might include loss of activity agianst IgG. There are fundamental differences between the two enzymes despite their shared substrate; SpeB is a broad spectrum protease with activities against a whole array of host proteins while EndoS is very specific for IgG. This might give a hint about their respeictive contribution to IgG hydrolysis. SpeB most likely contributes to IgG hydrolysis during infections but there will be many competing substrates that will lower the effeciency against IgG. It should also be mentioned, that *S. pyogenes *possesses another cysteine proteinase, IdeS, that in contrast to SpeB only hydrolyzes IgG [43]. EndoS on the other hand could at low concentrations efficeintly incapacitate IgG during certain stages of the infection. Our current model hypothesizes that EndoS most likely plays a minor role in animals without aquired immunity towards *S. pyogenes *and only comes into play when there are circulating antibodies towards the bacteria. We are currently setting up animal models to test this hypothesis. Animals will be immunized with surface structures from *S. pyogenes *prior to challenge with wild type bacteria and an isogenic mutant in the *ndoS *gene. This might help us to further elucidate the role for EndoS during infections.

## Conclusion

The cysteine proteinase SpeB processes, inactivates, and ultimately degrades the IgG glycan-hydrolyzing enzyme EndoS. Glutamic acid 235 in EndoS is required for glycan-hydrolyzing activity and tryptophans in EndoS are involved in the enzymatic activity on human IgG. This is important information for future studies of the function and presence of EndoS in various systems. Since SpeB and EndoS are expressed during the same conditions (*in vitro *and possibly during some conditions *in vivo*), researchers setting out to study EndoS are bound to experience degradation/inactivation of EndoS if not the temporal production and activity of both enzymes are taken into account.

## Methods

### Bacteria and growth conditions

The *S. pyogenes *strains that were used in this study are AP1 of serotype M1 from the WHO Collaborating Center for Reference and Research on Streptococci, Prague, Czech Republic; AL1, an isogenic mutant of AP1 lacking production of active SpeB generated as previously described [2,24]; MC14, an isogenic mutant of AP1 lacking production of EndoS generated as previously described [2]. For maximal SpeB and EndoS expression, C-medium (CM) was used consisting of 0.5% (w/v) Proteose Peptone No. 2 (Difco, Detroit, MI) and 1.5% (w/v) yeast extract (Oxoid, Basingstoke, England) dissolved in CM buffer (10 mM K_2_PO_4_, 0.4 mM MgSO_4_, and 17 mM NaCl pH 7.5) [21]. When appropriate, a final concentration of 5 mM dithiothreitol (DTT) was added to the growth medium to activate SpeB. Strains AL1 and MC14 were cultured in the presence of 200 μg/ml of kanamycin for selective pressure.

### Protein electrophoresis and Western blots

Culture supernatants were precipitated using trichloroacetic acid at a final concentration of 5% and separated by 10% SDS-PAGE [44] followed by transfer to PVDF membranes (Immobilon-P, Millipore, Bedford, MA) by electroblotting. Secretion of SpeB and EndoS was analyzed using Western blots with polyclonal rabbit antiserum raised against the zymogen form of SpeB and full-length EndoS as previously described [2,45]. Densitometric analysis of stained SDS-PAGE gels was performed using the public domain software ImageJ 1.39f developed by Wayne Rasband at the National Institutes of Health .

### Proteins and enzyme activity assays

Full-length EndoS with (GST-EndoS) or without (rEndoS) glutathione-S-transferase (GST) as a fusion partner was recombinantly expressed and purified from *Escherichia coli *harboring the plasmid pGEX*ndoS *as previously described [34]. The native zymogen form of SpeB was purified from *S. pyogenes *strain AP1 using ion-exchange chromatography as previously described [18]. The immunoglobulins used in all experiments were affinity purified pooled human polyclonal IgG (Sigma, St. Louis, MO). For testing of SpeB activity on EndoS, 30 μg of rEndoS was incubated for 2 hours at 37°C with 1, 0.2, 0.04, or 0.008 μg of purified SpeB in 20 μl PBS with a 10 mM final concentration of DTT followed by 10% SDS-PAGE analysis. For testing of thermolysin degradation 10 μg of rEndoS was incubated with 1 μg of thermolysin (Sigma) [46] for 1 hour at 37°C in 10 mM Tris-HCl (pH 7.4) and analyzed on SDS-PAGE as above. Alternatively, 30 μg of rEndoS was incubated at 37°C with a fixed amount of SpeB (6 μg) in a final volume of 120 μl under the same conditions as above and samples were withdrawn at 1 or 30 min, and 1, 2, 3 or 4 hours. Reactions were terminated by a final concentration of 100 μM E-64 (L-*trans-*epoxysuccinylleucylamido(4-guanidino)butane), a specific cysteine proteinase inhibitor [47] followed by 10%SDS-PAGE analysis. EndoS activity on IgG was measured by withdrawing 2 μl of samples from the above mixture and incubated it with 2 μg of purified human IgG for 2 hours at 37°C followed by separation by 10% SDS-PAGE, or electroblotted onto PVDF membranes (Millipore). Glycosylated IgG was detected using 5 μg/ml of biotinylated *Lens culinaris *agglutinin lectin (LCA) and 1 μg/ml of Streptavidin-Horseradish peroxidase (Vector Laboratories, Burlingame, CA) and SuperSignal West Pico peroxidase substrate (Pierce, Rockford, IL). Membranes were analyzed using a Chemidoc XRS imaging system and Quantity One image analysis software (Bio-Rad, Hercules, CA).

### Homology modeling

Amino acids 37–446 of EndoS was aligned with EndoF_3 _from *Elizabethkingia meningoseptica *(formerly *Flavobacterium meningosepticum*) [33] using the T-Coffee method [48] and submitted to the SWISS-MODEL automated protein homology server [49,50] using EndoF_3 _(PDB 1EOK) as the template. The generated model was visualized using the VMD 1.8.5 software [51] and high resolution images were generated using POV-Ray 3 [52] running on a Mac OS X workstation.

### Chemical modification of EndoS

Chemical modification of tryptophan residues was performed according to [37]. Briefly, 20 μg of rEndoS was incubated with 0.5 mM N-bromosuccinimide (NBS) (Sigma) in 20 μl of 0.1 M citric acid-Na_2_HPO_4_, pH 4.5 for 30 minutes at room temperature. Chemical modification of cysteine residues as performed by incubating 20 μg of rEndoS with 10 mM N-ethylmaleimide (NEM) (Sigma) in 20 μl of 0.1 M citric acid-Na_2_HPO_4_, pH 6 for 30 minutes at room temperature or with 20 mM iodoacetamide (IAA) (Sigma) in 20 mM Tris-HCl, pH 8.5 for 30 minutes at room temperature. For determination of enzymatic activity, 0.5 μg rEndoS from the above reaction mixtures was incubated with 10 μg human IgG in 20 mM Tris-HCl, pH 7.4 for 2 hours at 37°C. Samples were separated by 10% SDS-PAGE and stained with Coomassie or electroblotted onto PVDF for analysis with LCA lectin blot as above.

### Site-directed mutagenesis of EndoS

Mutation of glutamic acid 235 (Glu-235) into glutamine (E235Q) was performed using QuickChange II Site-Directed Mutagenesis Kit according to manufacturer's instructions (Stratagene, La Jolla, CA). The mutagenic oligonucleotide primers (mutation underlined) used was 5'-CCT TGA TGG CTT AGA TGT GGA TGT TCA ACA TGA TAG TAT TCC-3' for E235Q in combination with the anti-sense of the above sequences and the plasmid pGEX*ndoS *generating plasmid pGEX*ndoS*(E235Q). Mutation was verified by sequencing. Recombinant EndoS(E235Q) was expressed and purified as described above for EndoS.

## Authors' contributions

MA participated in the design of the study and performed the experiments. MC and AO conceived of the study and participated in its design. MC performed sequence alignments and homology modeling, and drafted the manuscript. All authors read and approved the final manuscript.
